# The Queen Bee phenomenon in Academia 15 years after: Does it still exist, and if so, why?

**DOI:** 10.1111/bjso.12408

**Published:** 2020-07-22

**Authors:** Klea Faniko, Naomi Ellemers, Belle Derks

**Affiliations:** ^1^ University of Geneva Switzerland; ^2^ Utrecht University The Netherlands

**Keywords:** gender discrimination, glass ceiling, Queen Bee phenomenon

## Abstract

Fifteen years ago, the British Journal of Social Psychology published a set of studies on male and female academics, documenting that female faculty members were more likely than male faculty members to express stereotyped views of women at the beginning of their academic careers (PhD candidates; Ellemers *et al*., 2004, *Br. J. Soc. Psychol*., *43*, 3). At the same time, the self‐descriptions of female faculty members were just as masculine as those of their male colleagues. Ellemers and colleagues (2004, *Br. J. Soc. Psychol*., *43*, 3) referred to this combination of results as indicating the existence of a ‘Queen Bee (QB) phenomenon’ in academia. The present contribution investigates whether the QB phenomenon is also found among current generations of academics, investigating this in two recent samples of academic professionals (*N* = 462; *N* = 339). Our findings demonstrate that the phenomenon first documented in 2004 still exists: Advanced career female academics are more likely than their male counterparts to underestimate the career commitment of women at the beginning of their academic careers. At the same time, both male and female academics at advanced career stages describe themselves in more masculine terms than those at early career stages. We argue this indicates a response pattern in which successful women emulate the masculinity of the work environment. To indicate this, the term ‘self‐group distancing’ might be more appropriate than ‘Queen Bee effect’.

## Background

I have the impression that my female doctoral students are spoiled. They are not available to work on evenings and the weekend. They are busy with their boyfriend. For my male doctoral students, the career is everything.^1^ Female professor

Reaching a fair representation of women in top level positions is a serious challenge for many organizations. It is often assumed that promoting some women into key positions will make it easier for other women to follow. But is it really as simple as that? Are successful women in male‐dominated professions and organizations indeed ready to support women in early career stages? Although fictional, the 2006 comedy drama *The Devil Wears Prada* suggests the opposite. As the title suggests, the female manager played by Meryl Streep turns out to be particularly harsh and demanding towards her female intern.

What could be seen as ‘entertaining fiction’ is actually uncomfortably close to reality in some organizations. Research in social psychology has examined the mechanisms that lead women in senior positions to underestimate the abilities and dedication of women at early career stages, in this way – sometimes unwittingly – putting up obstacles for other women aiming to climb the organizational ladder. Instead of assuming these ‘devils’ are individuals with flawed personalities or reveal how women generally prefer to behave, we examine the possibility that highly masculine organizations offer career experiences that encourage such responses in women. If this is the case, preventing such patterns is only possible when organizational cultures and images of success become more diverse and inclusive.

### What is the Queen Bee phenomenon?

Fifteen years ago, the British Journal of Social Psychology published a contribution in which differential career ambitions and stereotypical perceptions of male and female academics were examined as possible reasons for the underrepresentation of women in academia (Ellemers, van den Heuvel, de Gilder, Maass, & Bonvini, 2004). Ellemers *et al*. (2004) reported two studies conducted at universities in the Netherlands and Italy. While they observed no difference in the self‐stated ambitions of male and female PhD candidates, female faculty members were more likely than their male colleagues to underestimate the career ambitions of junior female academics. Further, female faculty members reported a gender identity that was equally masculine as their male colleagues (in Study 2). The authors argued that the tendency of female faculty members to *perceive* female PhD candidates as less committed to their career than male Phd candidates (while in actuality male and female PhD candidates *reported* similar career ambitions) while describing themselves in highly masculine terms would indicate how they fit the masculine prototype of the successful academic and were different from other women. They explained this phenomenon by arguing that these are all indicators of a response pattern relating to the career experiences of women in male‐dominated organizations.

The authors referred to these findings as the *Queen Bee phenomenon* (a term that was introduced by Staines, Tavris, & Jayaratne, 1974). They suggested that these responses can be seen as stemming from career advancement strategies that women in male‐dominated organizations use to contend with gender‐stereotypical expectations that underestimate the abilities and ambitions of women. This induces some women to emphasize their exceptional ambition and masculinity, which should separate them from (the stereotype of) other women, resulting in ‘self‐group distancing’. Ellemers *et al*. (2004) argued that: ‘…survival of women in a male‐dominated work environment entails a form of individual mobility, in the sense that they have to prove to themselves and others that they are unlike other women in order to be successful in an academic career’. (p. 333).

### What is the added value of repeating research conducted 15 years ago?

When Ellemers *et al*. (2004) conducted their initial studies, the female faculty members they examined represented a generation of women who constituted a small minority among academic faculty. In fact, although they consistently observed QB responses in both their studies (see Ellemers *et al*., 2004; Tables 1 and 2) follow‐up analyses of the main results in Study 2 revealed that this pattern of results was most clearly visible among the generation of women born between 1921 and 1949 (see Ellemers *et al.,* 2004; Tables 3 and 4). These were women who had started their academic careers when women's equal rights in the labour market still were contested. Many of them would have been be the first woman in history to enter a position on their faculty. Ellemers *et al*. (2004) argued that: ‘…these data are consistent with our theoretical argument that when it is exceptional for women to pursue an academic career, those who are successful in doing so perceive themselves as non‐prototypical members of their gender group’ (Ellemers *et al*., 2004, p. 332). However, they also noted that – due to the small number of older female faculty in their sample – this aspect of their analysis was inconclusive and in need of further research. Thus, Ellemers *et al*. (2004) reasoned that QB responses are caused by personal career experiences and speculated that these might no longer emerge in future generations of female academics. In fact, the final sentence of their paper reads: ‘ Thus, when it no longer seems necessary to distance oneself from other gender group members in order to prove one can be successful at work, this may prevent gender stereotypes from affecting the career opportunities of women at the university’. (p. 332).

For current generations of academics, the enrolment of female students in higher education is self‐evident and the presence of female faculty members is no longer exceptional. However, this does not necessarily imply that gender stereotypes no longer affect the career opportunities of women at the university. Indeed, we note that policies aiming to attract, advance, and retain women within universities and research institutions have not yet had the intended effects, and efforts towards achieving these goals have only increased during the last years (see League of European Research Universities, 2019). Further, recent examinations of different samples of professional women (which we review below) offer evidence that QB responses are still observed, and can be related to the negative career experiences that women still encounter during their careers in different professions and organizations (e.g., the police force) where higher power and status roles are still occupied mostly by men (Derks, Ellemers, Van Laar, & de Groot, 2011; Derks, Van Laar, Ellemers, & de Groot, 2011; Faniko, Ellemers, & Derks, 2016; Faniko, Ellemers, Derks, & Lorenzi‐Cioldi, 2017).

This is why we consider it of interest to examine the QB phenomenon once again among the current generation of female academics and compare the results obtained to those reported by Ellemers *et al*. (2004). This allows us to examine whether QB responses have disappeared with increasing representation of women in academia, or persist even among female academics today. In addition, we aim to understand whether – despite the presence of women – the organizational culture in academia might implicitly continue to define career success in terms of the masculine stereotype. To examine this, we also compare gender‐stereotypical self‐views of men and women at the beginning of their academic careers (PhD candidates) to those who are more advanced in their career (faculty members). This should inform our understanding of whether and how images of success in the academic environment are reflected in the self‐views of men and women at different career stages. Both these insights might benefit attempts to offer equal work conditions for men and women working in academic institutions.

### Scientific explanations for the Queen Bee phenomenon

Since its publication, the studies reported by Ellemers *et al*. (2004) have been referenced over 391 times by researchers in social psychology, economics and management, and organizational studies. The phenomenon has also inspired public discourse and multiple media reports over the years, focusing on the propensity of professional women to be highly competitive towards each other, and reluctant to support other women. Indeed, many media accounts selectively report their favoured interpretation of the academic literature on the Queen Bee phenomenon, claiming that rivalries between women are specific to the nature of women.

Such responses have also been observed by researchers working in different theoretical and research traditions, and are indicated with a variety of terms. For instance, *Tug of War* is a term used to describe female rivalry in the workplace (Williams, 2014; Williams, Berdahl, & Vandello, 2016; Williams & Dempsey, 2014). *Cat fights* is another term used in the field of communication sciences to describe the same female rivalry (Tanenbaum, 2002). Similar phenomena have been studied in different strands of academic research (see Cowan, Neighbors, DeLaMoreaux, & Behnke, 1998; Duguid, 2011; Etzkowitz, Kemelgor, Neuschatz, Uzzi, & Alonzo, 1994; Gabriel, Butts, Yuan, Rosen, & Sliter, 2018; Jones & Palmer, 2011; Markovits, Gauthier, Gagnon‐St‐Pierre, & Benenson, 2017; Mavin, 2008; Sheppard & Aquino, 2012a, 2014; Tanenbaum, 2002). In all cases, the terms chosen to describe these phenomena indicate hostile behaviour between women in the workplace. The recognition of this behaviour both in scientific research and in public media suggests that the phenomenon is (still) valid and widespread. At the same time, the terms used to indicate this response pattern not always accurately represent emerging insights about the origins and true nature of the QB phenomenon.

The first paper (Ellemers *et al*., 2004) on the Queen Bee phenomenon was the starting point of a broader research programme on the psychological effects of underrepresentation of women (see Derks, Van Laar, & Ellemers, 2016 for an overview). The relevance of this phenomenon has now been documented with empirical evidence obtained through different samples, with data collected in a variety of work environments and national contexts. More concretely, it has been examined among senior policewomen in the Netherlands (Derks, Ellemers, *et al*., 2011), among women in business leadership positions in the Netherlands (Derks, Van Laar, *et al*., 2011), Switzerland and Albania (Faniko *et al*., 2016, 2017), and in Indonesia (Permatasari & Suharnomo, 2019), among female professors in Italy and Spain (Bagues, Sylos‐Labini, & Zinovyeva, 2017), and among executives and senior female managers in South Africa (Johnson & Mathur‐Helm, 2011) as well as in the United States (Workplace Bullying Institute, 2010; for reviews, see Derks *et al*., 2016; Ellemers, Rink, Derks, & Ryan, 2012; Faniko, Chipeaux, & Lorenzi‐Cioldi, 2018).

In this literature, a growing body of evidence confirms the notion that QB responses are triggered by specific career experiences in male‐dominated work environments (Derks, ; Faniko *et al*., 2016, 2017). For instance, correlational data reveal that – depending on initial levels of gender identification – there is a relation between experiences of gender discrimination and QB responses. Specifically, QB responses were found mostly among women holding senior positions, who showed low gender identification at the beginning of their career and had experienced gender discrimination as their career advanced (Derks, Ellemers, *et al*., 2011). Further, experimental research additionally demonstrated that being reminded of gender bias triggered these responses, among women who identified weakly with their gender group at work (Derks, Van Laar, *et al*., 2011). However, these responses were not found when women were reminded of situations at work where they were judged on the basis of their individual merit. These findings suggest that career experiences *moderate* QB responses: These do not occur as a matter of course, but emerge when women suffer discriminatory career experiences, or when they consider these experiences. In fact, similar self‐group distancing responses were observed among cultural minorities (both men and women) who advanced to higher professional levels (Derks *et al*., 2015).

Other studies further contribute to the conclusion that QB responses are not characteristic of the way in which women generally approach a professional career. This research shows that such responses only become visible at senior career stages, and are related to the negative experiences encountered by women during the course of their career. Specifically, two studies showed that the increased tendency for women in managerial positions (compared to women in subordinate positions) to display QB responses was *mediated* by personal sacrifices they had made in different domains (family, personal convictions, vacation) to achieve career success (Faniko *et al*., 2017). This offers additional evidence that experiences that prompt women – more than men – to make personal sacrifices for success as they advance in their careers explain why women in managerial positions show QB responses.

Empirical research additionally shows that women report having received less organizational support than men do as they advance to higher organizational levels (Ellemers, 2014; Ellemers *et al*., 2012; Mulcahy & Linehan, 2014; Ryan & Haslam, 2007). The experience that – to be successful in their career – women have to make more sacrifices while receiving less support than men easily leads to the inference that this makes them different from women at early career stages, many of whom might not be equally motivated and persistent in making similar sacrifices to succeed in their career.

Together, these studies show that the response pattern seen to characterize the ‘Queen Bee’ is not to be attributed to ‘the way some women are’ or how they typically interact with each other at work. Instead, research reveals that factors in the organizational context and more specifically the exposure to gender‐stereotypical expectations, negative career experiences, and lack of organizational support contribute to the maintenance of the QB phenomenon.

### Does the Queen Bee phenomenon still exist in academia?

There is reason to believe that current generations of women in academia might no longer display QB responses. Since 2004, several actions have been undertaken by different institutions to promote women's careers in academia and increase female representation, including at the highest job levels. Various gender equality programmes and action plans have attempted to improve the conditions, and opportunities for women access, employment, and equal pay in academia (for reviews, see League of European Research Universities, 2019). The share of female students graduating from universities now exceeds that of male students and for women pursuing an academic career is not as exceptional as used to be the case for prior generations of female professors, many of which went through the experience of being the first woman ever having reached a faculty position in their field. The introduction of gender equality initiatives, as well as an increasing number of female students and researchers, implies that nowadays it is less exceptional than for previous generations of women to make a career as a female academic.

At the same time, there also is reason to believe that, despite the increased presence of women in the workplace including academia, stereotypes about women in science persist and impede their career progress (National Academies of Sciences, Engineering, & Medicine report, 2020). In fact, many women in academia still suffer sexism and negative career experiences that might trigger QB responses (Tenbrunsel, Rees, & Diekmann, 2019). Following up on the #MeToo movement, the US National Science Foundation (2018) and National Institutes of Health (2019) have recently noted that ongoing harassment and sexism in academia may discourage women from pursuing an academic career. Despite the larger influx of female students and junior academics, relevant statistics continue to indicate a so‐called scissor effect between the genders that persists through different cohorts of men and women. That is, even in disciplines where women have been overrepresented among PhD's and at early career stages for many years (such as psychology, Clay, 2017), they are still clearly underrepresented at higher job levels in academia, indicating their lesser likelihood of being promoted and higher dropout rate at each career stage (European Commission, 2019). Thus, regardless of the presence of gender equality programmes and the increasing number of female students and researchers, continuing indications of sexism and gender bias in academia suggest that the QB phenomenon may still be present today.

### The current research

The current research examined evidence for two competing hypotheses, explaining the QB phenomenon either from the career experiences of a specific generation of women (generation hypothesis), or from the experiences female academics still encounter today while advancing in their career (academic experience hypothesis). *The generation hypothesis* was advanced by Ellemers *et al*. (2004) as a possible explanation for their effects, and is based on the knowledge that the career success of female academics nowadays is less exceptional than it was for the sample of women examined by Ellemers *et al*. (2004). Due to implementation of gender equality programmes and the increasing number of female researchers, it might be that the QB phenomenon is no longer visible, when comparing the data from the Ellemers *et al*. (2004) paper (prior generation of female academics) with the present results (current generation of female academics).

*The academic experience hypothesis* is based on evidence reviewed above, suggesting that sexism and gender discrimination persist within universities and research institutions despite increasing numbers of female academics present. If women today are still exposed to these negative career experiences, it is quite possible that the QB phenomenon is still present. This would imply that even in current generations women continue to be exposed to a masculine organizational culture as they advance in their career. This might then elicit QB responses, resulting in differences between the way men and women at advanced career stages view male and female early career stage academics. To examine this possibility, in the current data we will compare how women and men at advanced academic career levels (1) perceive early career female and male academics and (2) how men and women describe themselves in masculine terms at early versus later career stages, by comparing self‐views of male and female doctoral students with those of male and female faculty members.

To examine support for each of these hypotheses, we repeated the research reported by Ellemers *et al*. (2004), to examine evidence for the QB phenomenon in two recent samples of academics. More concretely, like Ellemers *et al*. (2004) did, we compared how female and male faculty members perceived the career commitment of male and female PhD candidates and assessed whether male and female PhD candidates and faculty members described themselves in stereotypically masculine terms. This also parallels our recent research (Derks, Ellemers, *et al*., 2011; Derks, Van Laar, *et al*., 2011; Faniko *et al*., 2016, 2017), in which we compared perceptions and self‐views of men and women at different career stages in other employment contexts. The comparison of the results reported by Ellemers *et al*. (2004) with the current results allows us to examine support for the generation hypothesis. The comparison between male and female participants at different career stages in the current research allows us to examine support for the academic experience hypothesis.

## Methods

### Participants

The two data sets^2^ reported in the current research were collected in Switzerland. The sample of Study 1 consisted of 462 participants (age 23–68, *M* = 39.21, *SD* = 12.23), all academics employed at one of the nine different faculties and interfaculty institutions at a large university. Of them, 248 were PhD candidates (166 female and 82 male, age *M* = 29.28, *SD* = 3.65), which we consider as early career academics, and 211 were (tenured) faculty members in lecturing and professorship positions (85 women and 126 men, age *M* = 50.79, *SD* = 7.69), which we consider as advanced career academics. The group of faculty members consisted of senior lecturers/maîtres d'enseignement et de recherché (18%), assistant professors/professeurs assistant (18.5%), adjunct professors/professeurs titulaires (6.2%) associate professors/professeurs associés (27%), and full professors/professeurs ordinaires (30.3%). Post‐docs and other academic staff on temporary and/or part‐time contracts were not considered, as these constitute a very heterogeneous group both in terms of career stage and involvement in academic life.

The sample of Study 2 consisted of 339 scientists (*M* = 34.98, *SD* = 9.69) working in STEM disciplines at an internationally oriented academic research institute. In terms of career stage, there are 193 participants (63 women and 130 men, age *M* = 29.01, *SD* = 3.39) including junior research fellows and PhD candidates that we consider as early career academics, and 146 participants (36 women and 110 men, age *M* = 42.86, *SD* = 9.68) who have managerial responsibilities; we consider these as advanced career academics.

In terms of representing academics at different career stages, the current samples resemble the samples examined by Ellemers *et al*. (2004). In both cases, these are early career academics (mostly PhD candidates) and advanced career academics (tenure track or tenured faculty members) working at a large academic institution in Europe^3^.

### Measures

#### Career commitment

As in the research conducted by Ellemers *et al*. (2004), the *questionnaire for the early career academics* comprised a scale measuring self‐reported career commitment (Ellemers, De Gilder, & Van den Heuvel, 1998). Early career participants were asked to report their personal career commitment on four items ranging from 1 (strongly disagree) to 7 (strongly agree) (Study 1, α = .86; Study 2, α = .81): ‘Professional success is one of the most important things in my life’, ‘I often think about what I can do to get ahead in my career’, ‘Most of my ambitions have to do with my career’, and ‘My career plays a central role in my life’. The same items were adjusted to measure the perceived career commitment of male (Study 1, α = .94; Study 2, α = .91) and female PhD candidates (Study 1, α = .91; Study 2, α = .90). The *questionnaire for the advanced career academics* consisted of a scale assessing perceived levels of career commitment among junior academics. The only difference was that while in the Ellemers *et al*. (2004) research participants were randomly asked either to rate typical female doctoral or typical male doctoral students, in the current research the statistical power was increased by asking each participant to rate both typical male and female doctoral students.

#### Self‐reported masculinity

As in the studies conducted by Ellemers *et al*. (2004), the items capturing self‐perceived masculinity were based on Bem's Sex Role Inventory (Bem, 1974). We assessed the extent to which early career and advanced career academics perceived themselves in masculine terms. In addition to items that focused on agency (independent, have leadership qualities, willing to take risks), we added two traits to represent the aspect of assertiveness in the masculine stereotype (self‐confident, willing to take the initiative; Abele, Uchronski, Suitner, & Wojciszke, 2008; Diekman & Eagly, 2000).^4^ Both groups of academics were asked to indicate to what degree stereotypically masculine traits (characterized themselves (α = .73, both studies).

#### Background variables

Participants reported their background characteristics such as gender, age, nationality, their position, marital status, and number of children.

## Results

### Are female academics at early career stages less committed to their career than men?

**Figure 1:**
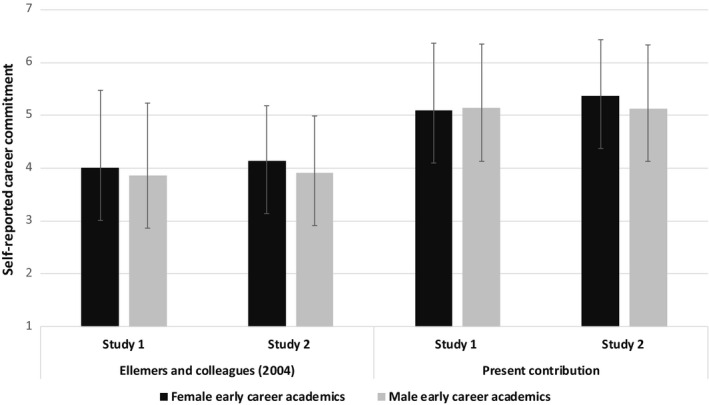
Self‐reported career commitment of female and male early career academics.

As reported by Ellemers *et al*. (2004), an analysis of the self‐reported career commitment of early career academics did not reveal evidence for a difference between female and male early career academics^5^ in either sample, Study 1: *F*(1, 246) = 0.05, *p* = .816, $\eta_{p}^{2}$ = .00, Study 2: *F*(1, 191) = 1.93, *p* = .166, $\eta_{p}^{2}$ = .01, see Figure 1.

### Who has stereotypical expectations about female early career academics?

A repeated measures MANOVA with the perceived career commitment of targets (male vs. female early career academics) as within‐participant variable, and participants’ gender and career stage (early vs. advanced career academics) as between‐participant variables was conducted to examine whether the career commitment of female early career academics was perceived as different from that of male early career academics, and if so by whom. Results revealed a significant two‐way interaction between target gender and participant's gender on perceived career commitment, *F*(1, 455) = 8.07, *p* = .005, Wilks's Λ = .98, $\eta_{p}^{2}$ = .02 (Study 1) and between target gender and participant's career stage, Study 1: *F*(1, 455) = 7.64, *p* = .006, Wilks's Λ = .98, $\eta_{p}^{2}$ = .02; Study 2: *F*(1, 335) = 4.50, *p* = .003, Wilks's Λ = .99, $\eta_{p}^{2}$ = .01. In both studies, these effects were qualified by a three‐way interaction between participant's gender, target gender, and participant's career stage, Study 1: *F*(1, 455) = 9.28, *p* = .005, Wilks's Λ = .98, $\eta_{p}^{2}$ = .02; Study 2: *F*(1, 335) = 4.26, *p* = .004, Wilks's Λ = .99, $\eta_{p}^{2}$ = .01.

**Figure 2:**
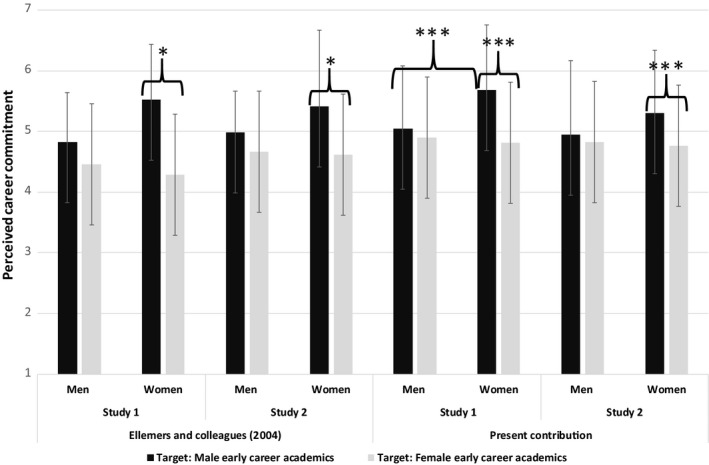
Perceived levels of career commitment of female and male early career academics among female and male advanced career academics. Note: *p < .05; **p < .01; ***p < .001.

In line with the QB response pattern and consistent with results reported by Ellemers *et al*. (2004), female advanced career academics perceived female early career academics as less career committed than male early career academics (*p* < .001, both studies), while this was not the case for male advanced career academics (all *ps* ns; see Figure 2, and Table S1). The ratings provided by female advanced career academics also differed from those offered by female early career academics (*p* = .002, *p* < .001, Study 1, Study 2, respectively), while men, regardless of their career stage, did not show evidence of gender stereotyping (all *ps* ns).

### What are the gendered self‐views of academics at different career stages?

**Figure 3:**
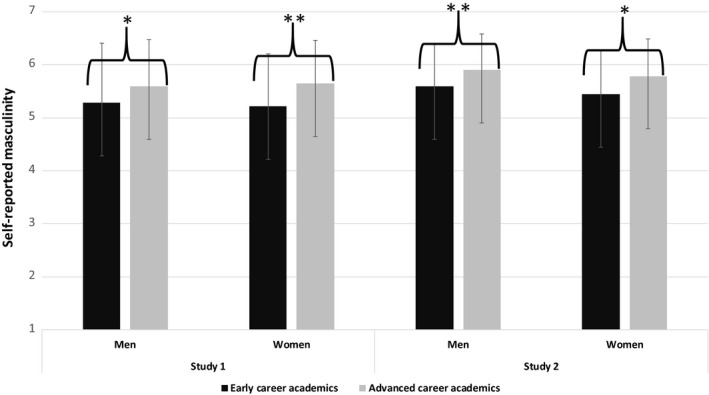
Self‐reported masculinity of academics as a function of participant gender and career stage (early vs. advanced career academics). Note: *p < .05; **p < .01; ***p < .001.

A 2 (male vs. female participants)‐by‐2 (early vs. advance career stage) between‐participants analysis of variance on self‐reported masculinity only revealed a main effect of career stage, *F*(1, 455) = 14.97, *p* < .001, $\eta_{p}^{2}$ = .03; *F*(1, 335) = 12.16, *p* = .005, $\eta_{p}^{2}$ = .03 (Study 1, Study 2, respectively) indicating that both female and male academics at advanced career stages offered more masculine self‐descriptions than female and male academics at early career stages (see Figure 3). That is, as reported by Ellemers *et al*. (2004, Study 2, p. 330), the masculine self‐descriptions observed among female advanced career academics are not reliably different from those of their male colleagues (*M*_women_ = 5.65, *SD* = 0.82; *M*_men_ = 5.59, *SD* = 0.89; *M*_women_ = 5.79, *SD* = 0.70; *M*_men_ = 5.90, *SD* = 0.69; *p* = .653, *p* = .459, Study 1, Study 2, respectively).

In the present research, we additionally examined self‐perceived masculinity of male and female researchers at early career stages. This allows us to see that female advanced career academics indicated higher levels of masculinity in their self‐reports than female early career academics, suggesting increased self‐group distancing as they advanced in their career (*M*_advanced career academics_ = 5.65, *SD* = 0.82; *M*_early career academics_ = 5.22, *SD* = 0.99, *M*_advanced career academics_ = 5.79, *SD* = 0.70; *M*_early career academics_ = 5.45, *SD* = 0.83, *p* = .001, *p* = .032, Study 1, Study 2, respectively). However, we note that the same pattern was also observed among men, indicating they too perceive themselves as more masculine as they advance in their career (*M*_advanced career academics_ = 5.59, *SD* = 0.89; *M*_early career academics_ = 5.29, *SD* = 1.12, *M*_advanced career_ = 5.90, *SD* = 0.69; *M*_early career academics_ = 5.59, *SD* = 0.80, *p* = .001, *p* = .032, *p* = .033, *p* = .002, Study 1, Study 2, respectively). Further, no significant difference was observed among female and male early career academics (*p* = .540, *p* = .225, Study 1, Study 2, respectively).

## Discussion

The goal of the present research was to test whether the QB phenomenon, first reported by Ellemers *et al*. (2004), can still be observed in academic settings today. Our data offer no support for the ‘generation hypothesis’, advanced by Ellemers *et al*. (2004). That is, despite increasing presence of women in academia, the same pattern of results that was described 15 years ago is still visible in the two recent samples of academics reported here. In both our current samples, female advanced career academics hold stereotypical views of female early career academics. That is, whereas female and male early career academics indicate similar levels of career commitment in their self‐reports, female advanced career academics women *perceived* female early career academics to be reliably less dedicated to their career than men at early career stages. By contrast, male academics at advanced career stages did not perceive a difference between the career commitment of men and women at early career stages.

Even if the effect sizes observed here are relatively modest, the robustness of this pattern is evident from the similarity of results obtained across our two current samples, which are somewhat different in terms of the age range of participants, characteristics of the institutions and their tasks and job types, as well as their disciplinary representation, which covers the broad range of academic disciplines in Study 1 and focuses on STEM disciplines only in Study 2. Further, these findings also resonate with similar observations among women at advanced career levels in a range of different work contexts and job types, including commercial businesses as well as public organizations (e.g., Derks *et al*., 2016; Faniko *et al*., 2017).

Further, our findings do offer support for the ‘academic experience hypothesis’ as an explanation for the observed response patterns. That is, comparing male and female academics at different career stages indicates that both male and female academics offer more masculine self‐descriptions at advanced career stages than they do at early career stages. The present results do not allow us to determine whether this results from changes in the self‐views of women during the course of their career, or from the (self‐)selection of women with the most masculine self‐views. In both cases, the net result is that women at advanced career stages are more inclined to describe themselves as non‐prototypical group members (i.e., in masculine terms) than do women at early career stages. We note that in both these samples men too show more masculine self‐descriptions at advanced career stages than in their early career. The tendency for men as well as women to perceive themselves as more masculine at more advanced career levels does not seem incidental. Instead, this also is a pattern that has been documented before, in studies conducted at Dutch universities (Derks, van Veelen, & Handgraaf, 2018) and in a business context (Faniko *et al*., 2016). These results suggest that the organization equates masculinity with career success, implicitly communicating that – like men – women can only successful when they present themselves as stereotypically masculine. However, in the case of women this also implies that they self‐describe as non‐prototypical group members.

On the one hand, an organizational context that invites and rewards masculinity may seem less problematic for men than for women (Derks *et al*., 2018). On the other hand, we think women as well as men lose out if the organization only recognizes and rewards a specific model of success, making it less likely for individuals (M/F) with more feminine behavioural styles (e.g., focusing on cooperation and maintenance of a team atmosphere) to see how they might advance to a position of leadership. This points to a more general concern that has been documented in the context of diversity programs where individuals with different contributions and abilities are recruited and hired, but diversity benefits are lost because they either have to ‘fit in’ or ‘opt out’ (Ellemers, 2014; Ellemers & Rink, 2016). This also points to the type of initiative that would be needed to reduce the persistence of QB responses. Instead of encouraging more gender diversity by focusing on the *numeric* representation of women at different academic career stages, such initiatives should also aim to modify the homogeneously masculine organizational culture to make it more inclusive for different types of men and women (see also Şahin, Van der Toorn, Jansen, Boezeman, & Ellemers, 2019).

In sum, the current results suggest that the effects reported in 2004 still offer a valid account of how career experiences of women in academia contribute to the emergence and persistence of QB responses. The hope of Ellemers *et al*. (2004) was that future generations of academic women would be less likely to have such experiences, as they accessed the labour market after gender equality was enshrined in the law and the presence of women in institutions for higher education had become self‐evident. This hope is refuted by the present data. Instead, they fuel the concern also expressed by Ellemers *et al*. (2004), namely that low estimates of the academic ambitions of women at early career stages would not be recognized as stereotypical thinking as long as these were expressed by senior women (instead of men). Indeed, it turns out that men continue to be significantly more successful in climbing up the academic ladder (Beeler *et al*., 2019; European Commission, 2018). Despite a comparable professional performance, women are much more likely to drop out at some point in their career. In sum, it seems that – unless changes in the academic culture can be realized – the visible presence of senior women might harm rather than help the career success of younger women.

This research thus corroborates results from other studies, suggesting that the QB phenomenon is not a cause, but rather the *consequence* of gender discrimination that continues to prevail in academia, as in many other professional settings (Biggs, Hawley, & Biernat, 2018; Britton, 2017; Burke, 2017; Kuchynka *et al*., 2018; Maranto & Griffin, 2011). At the same time, the presence of women at advanced career levels, and efforts to implement policies that aim support the careers of women in academia, makes it less likely that continued discrimination is acknowledged. Instead, these tend to be seen as signalling that women and men nowadays have equal opportunities, or that women are in need of additional support because they are less competent than men (for reviews, see Dover, Kaiser, & Major, 2019; Ellemers, 2018). This strong belief in supposedly gender‐blind meritocratic principles, in fact, preserves current practices in academia which tend to value and reward stereotypically masculine and agentic traits (see also Derks *et al*., 2018). Despite the fact that this discourse is not actively promoted in formal guidelines, its prevalence in informal statements and behaviours should not be underestimated. At the same time, this culture also promotes the idea that personal sacrifices have to be made as a necessary condition to be a successful academic. As illustrated by the quote at the outset of this paper, female academics who have managed to advance in their careers despite these difficulties, by renouncing a series of typically female behaviours and life choices – including motherhood – may see this a necessary condition to be successful in academia. They may find it difficult to accept that younger colleagues expect to be equally successful without having to make such personal sacrifices (Faniko *et al*., 2017).

### Conclusion

The data presented here demonstrate that the QB phenomenon still exists 15 years after this phenomenon was documented in academia. These findings counter the generation hypothesis and support the academic experience hypothesis, as they suggest that women still need to adjust themselves to the masculine academic culture in order to advance in their career.

Is it still appropriate then to use the term Queen Bee to describe this phenomenon? We argue that the term QB phenomenon is in need of revision, as it suggests women are the problem, and should somehow be ‘fixed’. In an interview with The Atlantic magazine (Khazan, 2017), Carol Tavris, who was part of the research team that coined the expression QB phenomenon in 1974 (Staines *et al*., 1974) likewise regretted that their findings ‘had since been misinterpreted, carved into a cudgel for bashing women. If women are their own worst enemies, after all, why should people push for women's workplace advancement? She regrets that giving “a catchy name” to a complex pattern of behavior helped launch queen‐bee‐ism as “a thing” – one that has endured despite all the gains working women have made since the 1970s’.

In recent years, we have started to use the term ‘self‐group distancing’ to refer to the process by which women and other minorities may emphasize how they are different from their negatively stereotyped group in order to increase individual success (see, e.g., Derks *et al*., 2016; Van Veelen, Veldman, Van Laar, & Derks, forthcoming; Veldman, Meeussen, Van Laar, & Lo Bleu, 2020). As previous research (Derks, Ellemers *et al.,* 2011; Derks, Van Laar *et al.,* 2011; Faniko *et al.,* 2017) suggests the tendency to self‐distance from other women in the workplace is not a ‘natural tendency’ of women, but is a behavioral strategy prompted by (implicit) organizational definitions of success that are couched in masculine terms. It also conveys that efforts to increase diversity at academic institutions should not just focus on increasing the numbers of women (or other minorities), but aim to make these institutions more inclusive by rewarding more heterogeneous models of success, so that emphasizing masculine qualities and distancing from one’s group is no longer the royal road to success.

## Conflicts of interest

All authors declare no conflict of interest.

## Author contributions

Klea Faniko (Conceptualization; Data curation; Formal analysis; Investigation; Methodology; Project administration; Resources; Validation; Visualization; Writing – original draft; Writing – review & editing); Naomi Ellemers (Conceptualization; Data curation; Funding acquisition; Investigation; Methodology; Project administration; Resources; Supervision; Validation; Visualization; Writing – original draft; Writing – review & editing); Belle Derks (Conceptualization; Data curation; Investigation; Methodology; Resources; Validation; Visualization; Writing – original draft; Writing – review & editing).

## Supporting information


**Table S1.** Perceived levels of career commitment of female and male early career academics as a fonction of participant gender and career stage (early vs. advanced career academics).

## Data Availability

Data available on request due to privacy/ethical restrictions: The data that support the findings of this study are available on request from the corresponding author. The data are not publicly available due to privacy or ethical restrictions.

## Footnotes

^1^ This quote comes from a series of interviews conducted in the framework of Study 1, which was part of a larger research project.

^2^ These data sets were collected in the context of a larger study project, focusing on participant narratives and other interview and survey data, aiming to inform the Diversity Offices of these institutions about the impact of their gender equality initiatives. Only measures relevant to our specific research questions are reported here – these results are not reported elsewhere.

^3^ As a reminder, in the research reported by Ellemers *et al*. (2004), two groups of participants were recruited for Study 1 (the Netherlands) as well as Study 2 (Italy). In both studies, participants included scholars from all faculty departments of the university (Study 1: 13 departments; Study 2: 26 departments). Early career academics were all PhD candidates (Study 1, *N* = 132, mean age = 29; Study 2, *N* = 80, mean age = 28), and advanced career academics were all (tenured) faculty members (Study 1, *N* = 156, mean age = 47; Study 2, *N* = 93, mean age = 45). The second group comprised (the national equivalent of) assistant (Study 1: 49%, Study 2: 30%), associate (Study 1: 15%, Study 2: 34%), and full professors (Study 1: 36%, Study 2: 36%). Please note that in both these national academic systems, there is no natural progression to a higher job level after a number of years. At each level, faculty members can stay in the same position until they retire, unless they successfully apply for a vacant position at a different level.

^4^ An additional analysis only examining the four items indicating agency produced the same pattern of results.

^5^ Female early career academics (Ellemers *et al*., 2004, Study 1: *M* = 4.01, *SD* = 1.37; Study 2: *M* = 4.14, *SD* = 1.05; present data Study 1: *M* = 5.10, *SD* = 1.27; Study 2: *M* = 5.38, *SD* = 1.05); and male early career academics (Ellemers *et al*., 2004, Study 1: *M* = 3.86, *SD* = 1.33; Study 2: *M* = 3.92, *SD* = 1.07; present contribution, Study 1: *M* = 5.14, *SD* = 1.22; Study 2: *M* = 5.13, *SD* = 1.21).
