# Supplementary material for: The Queen Bee phenomenon in Academia 15 years after: Does it still exist, and if so, why?
Source: Br J Soc Psychol. 2020 Jul 22;60(2):e12408. doi: 10.1111/bjso.12408 (PMC8246980; doi:10.1111/bjso.12408)
Supplement: Supplementary file 1 — Table S1. Perceived levels of career commitment of female and male early career academics as a fonction of participant gender and career stage (early vs. advanced career academics). [file BJSO-60-383-s001.docx]

**Table 1.** Perceived levels of career commitment of female and male early career academics as a fonction of participant gender and career stage (early vs. advanced career academics)

|  |  | Ellemers and colleagues (2004) | | | | Present contribution | | | |
| --- | --- | --- | --- | --- | --- | --- | --- | --- | --- |
|  |  | Study 1 | | Study 2 | | Study 1 | | Study 2 | |
|  |  | Target | | | | | | | |
|  |  | Female early career academics | Male early career academics | Female early career academics | Male early career academics | Female early career academics | Male early career academics | Female early career academics | Male early career academics |
| Male perceivers | Early career academics |  |  |  |  | 5.19 (1.10)^a^ | 5.00 (1.16)^a^ | 5.14 (1.07)^a^ | 5.03 (1.14)^a^ |
|  | Advanced career academics | 4.83^ab^ (.81) | 4.46^ab^ (.85) | 4.99^ab^ (.67) | 4.67^ab^ (.95) | 5.05 (1.03)^a^ | 4.90 (1.18)^a, b^ | 4.95 (1.22)^a^ | 4.83 (1.12)^a, b^ |
|  |  |  |  |  |  |  |  |  |  |
| Female perceivers | Early career academics |  |  |  |  | 5.39 (1.32)^a, c^ | 5.23 (1.01)^a^ | 5.35 (.95)^a^ | 5.31 (.91)^a^ |
|  | Advanced career academics | 5.52^b^ (.92) | 4.29^b^ (.65) | 5.41^a^ (1.26) | 4.62^b^ (1.02) | 5.68 (1.08)^c^ | 4.81 (.98)^b^ | 5.30 (1.04)^a^ | 4.77 (1.03)^b^ |

Note: Means with different superscripts differ significantly in a between and within-participants comparison (ps < .05). All comparisons made with pairwise comparisons tests.
